# The thrombotic risk in Cushing’s syndrome—questions, answers, and the algorithm to consider in its assessment: part I—thrombotic risk not related to surgery

**DOI:** 10.3389/fendo.2024.1350010

**Published:** 2024-03-11

**Authors:** Agata Hanna Bryk-Wiązania, Mari Minasyan, Renata Świątkowska-Stodulska, Anetta Undas, Alicja Hubalewska-Dydejczyk, Susan M. Webb, Elena Valassi, Aleksandra Gilis-Januszewska

**Affiliations:** ^1^Chair and Department of Endocrinology, Jagiellonian University Medical College, Kraków, Poland; ^2^Department of Endocrinology, Oncological Endocrinology and Nuclear Medicine, University Hospital, Kraków, Poland; ^3^Department of Endocrinology and Internal Medicine, Faculty of Medicine, Medical University of Gdańsk, Gdańsk, Poland; ^4^The John Paul II Hospital, Kraków, Poland; ^5^Department of Thromboembolic Disorders, Institute of Cardiology, Jagiellonian University Medical College, Kraków, Poland; ^6^Department of Endocrinology, Hospital S Pau, Barcelona, Spain; ^7^Sant Pau Biomedical Research Institute (IIB-Sant Pau), Research Center for Pituitary Diseases, Barcelona, Spain; ^8^Centre for Biomedical Network Research on Rare Diseases (CIBERER) Unit 747, Instituto de Salud Carlos III, Madrid, Spain; ^9^Department of Medicine, Universitat Autònoma de Barcelona, Barcelona, Spain; ^10^Servicio de Endocrinología, Hospital e Institut de Recerca Germans Trias i Pujol, Badalona, Barcelona, Spain; ^11^Universitat Internacional de Catalunya (UIC), Barcelona, Spain

**Keywords:** Cushing's syndrome, ectopic adrenocorticotropic hormone syndrome, venous thromboembolism, thrombotic risk, thromboprophylaxis

## Abstract

**Introduction:**

Recently, it has been reported that there is a great diversity in strategies used for thromboprophylaxis in patients with Cushing’s syndrome (CS). An aim of this review was to discuss these practices in light of the existing data on the thrombotic risk in patients with CS and guidelines for medically ill patients.

**Methods:**

The four relevant topics and questions on thrombotic risk in CS were identified. The current guidelines on prevention and diagnosis of venous thromboembolism (VTE) were reviewed for the answers. An algorithm to consider in the assessment of the thrombotic risk in patients with CS was proposed.

**Results:**

To address both generic and CS-specific risk factors for VTE, the algorithm includes the stepwise approach consisting of Padua Score, urine free cortisol, and CS-VTE score, with no indication for routine thrombophilia testing in the prediction of an index VTE episode. Having confirmed VTE, selected patients require thrombophilia testing to aid the duration of anticoagulant treatment. The separate part of the algorithm is devoted to patients with ectopic adrenocorticotropic hormone syndrome in whom exclusion of VTE precedes introducing routine thromboprophylaxis to prevent VTE. The cancer-related VTE also prompts thromboprophylaxis, with the possible vessel invasion. The algorithm presents a unifactorial and multifactorial approach to exclude high-bleeding risks and safely introduce thromboprophylaxis with low-molecular-weight heparin.

**Summary:**

Our article is the first to present an algorithm to consider in the thrombotic risk assessment among patients with Cushing’s syndrome as a starting point for a broader discussion in the environment. A plethora of factors affect the VTE risk in patients with CS, but no studies have conclusively evaluated the best thromboprophylaxis strategy so far. Future studies are needed to set standards of care.

## 1 Introduction

Cushing’s syndrome (CS) is a rare disease with an incidence ranging from 0.7 to 2.4 per million people per year (1). The recent meta-analysis of 22 observational studies involving 6,537 patients by Wagner et al. demonstrated that CS is associated with an almost 18-fold higher risk of venous thromboembolism [VTE; odds ratio (OR) 17.8, confidence interval 15.24–20.85] when compared to the general population with similar demographic characteristics, which corresponds with a prevalence of VTE and pulmonary embolism (PE) of 3.2% and 0.95%, respectively (2). Such a prevalence was observed among patients at a mean age of 42.1 years, with a proportion of women of 76.2%, with a mean ( ± standard deviation, SD) body mass index (BMI) of 29.3 ± 1.7 kg/m^2^. Most of the patients (86.4%) had CS of pituitary origin and had a baseline urine-free cortisol of 7–8 times above the upper limit of normal (ULN) (2). The previous meta-analysis by van Zaane et al. published in 2009, which included eight observational studies involving 476 patients with CS, also showed increased thrombotic risk, with a prevalence of VTE ranging from 1.5% to 2.9% for VTE not provoked by surgery and from 0% to 5.6% for VTE provoked by surgery (3).

The increased risk of VTE in CS is multifactorial, but most experts have linked it with increased activity of factor VIII and von Willebrand factor (vWF), along with impaired fibrinolysis as a consequence of its enhanced inhibition, as evidenced by elevated blood levels of plasminogen activator inhibitor 1, thrombin-activatable fibrinolysis inhibitor, and α2-antiplasmin (4). Other potential mechanisms that might contribute to a prothrombotic state in CS involve hyperactivity of platelets represented by increased thromboxane B2 concentrations, increased thrombin generation as shown by elevated thrombin–antithrombin complexes, and increased fibrinogen level (4). The compensatory elevation of the natural anticoagulants such as protein C, protein S, and antithrombin only adds complexity to the prothrombotic phenomena in CS (4).

Recently, it has been reported that the current clinical practices for thromboprophylaxis management in patients with CS differ across the reference centers of the European Reference Network on Rare Endocrine Conditions (Endo-ERN) (5). Of note, the thromboprophylaxis protocol for patients with CS was provided by one of 25 surveyed centers (5). The strategies to identify patients most likely to benefit are still being developed (6). Thromboprophylaxis can decrease the incidence of postoperative VTE in patients with CS, as reported in two retrospective studies (7, 8). However, the most recent meta-analysis showed that the risk of VTE associated with the surgical management of patients with CS is lower than in hip surgery, the latter being associated with routine thromboprophylaxis (2). Prior to the surgery, patients with CS are frequently hospitalized in the non-surgical ward for diagnostic evaluation (9). Moreover, one-third of the thrombotic episodes (when considering both arterial and venous) occur prior to the surgery (10). Consequently, the decision about thromboprophylaxis associated with surgery may not be a simple “yes-or-no” question, and the stepwise decision-making process may be more accurate, starting already in the non-surgical ward.

The aim of this review was to discuss the current practices of thromboprophylaxis in light of the existing guidelines and to develop the algorithm to consider in the assessment and management of the thrombotic risk in patients with CS.

## 2 Methods

The authors identified four discrete topics that related to the current practices in the thromboprophylaxis management in patients with CS, presented recently (5): risk factors for VTE, thrombophilia, subtypes of CS, and low-molecular-weight heparin (LMWH). The current practices were presented, followed by the complimentary questions. The responses were based on the current guidelines for patients with CS (6, 9, 11, 12) and guidelines on the thromboprophylaxis and diagnosis of VTE for the medical inpatients developed by American Society of Hematology (13, 14), American College of Chest Physicians (15–18), American Society of Clinical Oncology (19), European Society of Cardiology (ESC) (20, 21), European Society for Vascular Surgery (22), British Society of Hematology (23), International Initiative on Thrombosis and Cancer (24), and International Society of Thrombosis and Hemostasis (25, 26). The Medline database was searched for the following search terms: “D-dimer Cushing’s syndrome,” “Ectopic Cushing’s syndrome venous thromboembolism,” and “Adrenocortical carcinoma venous thromboembolism,” “Pasireotide venous thromboembolism,” “Ketokonazole venous thromboembolism,” “Metyrapone venous thromboembolism,” “Cushing’s syndrome factor V Leiden,” “Cushing’s syndrome prothrombin mutation,” “Cushing’s syndrome antithrombin deficiency,” “Cushing’s syndrome protein S deficiency,” “Cushing’s syndrome protein C deficiency,” “Cushing’s syndrome von Willebrand factor gene promoter.” No statistics was performed. The comments and improvements to the suggested algorithm are expected at agata.bryk@uj.edu.pl.

## 3 Results

**Figure 1:**
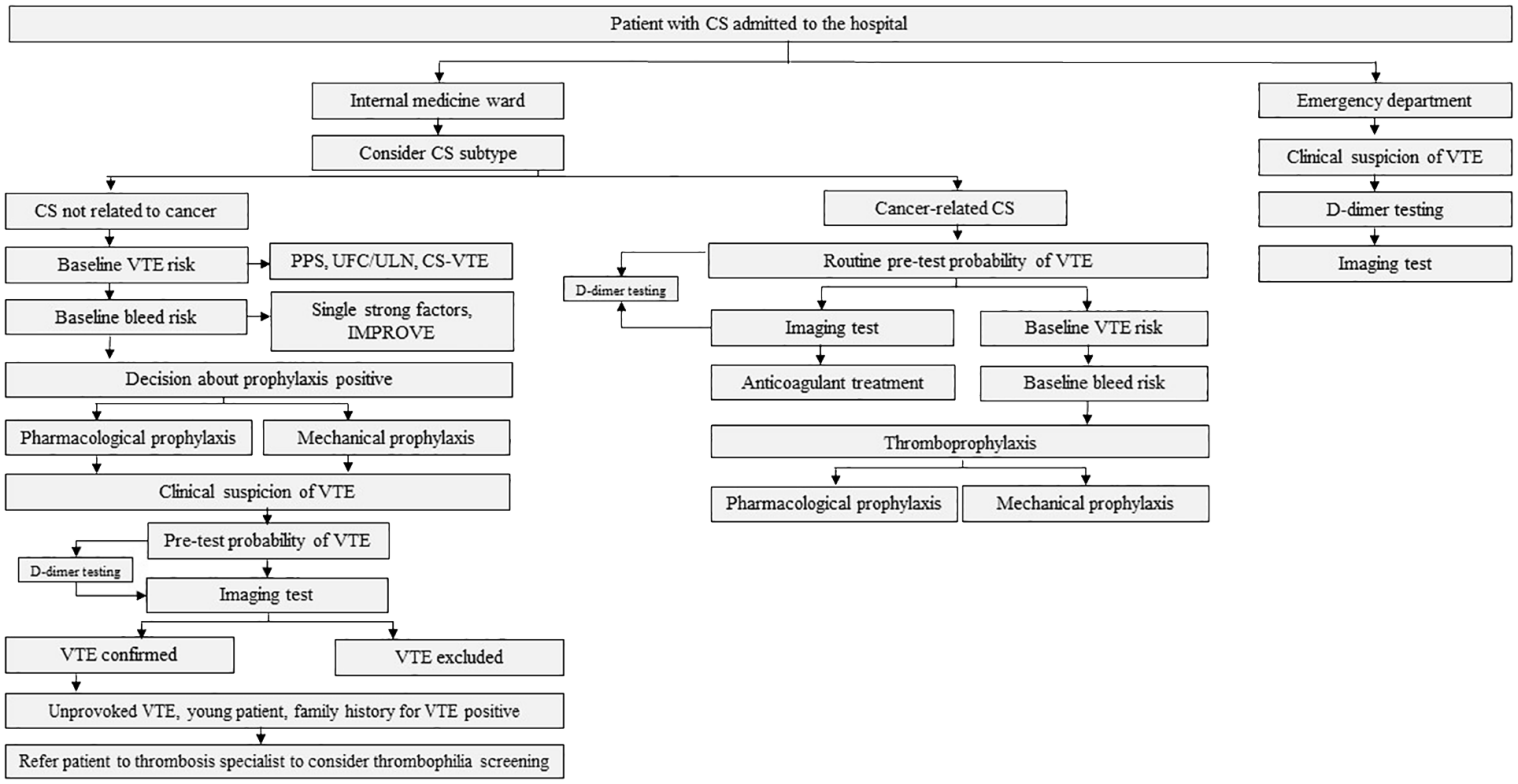
The algorithm for the thrombotic risk assessment in patients with Cushing’s syndrome (CS). VTE denotes venous thromboembolism; PPS, Padua Prediction Score; UFC, urine free cortisol; ULN, upper limit of normal; CS-VTE, Cushing Syndrome Venous Thromboembolism score; IMPROVE, International Medical Prevention Registry on Venous Thromboembolism; EAS, ectopic adrenocorticotropic hormone syndrome; ACC, adrenocorticotropic carcinoma.

**Figure 2:**
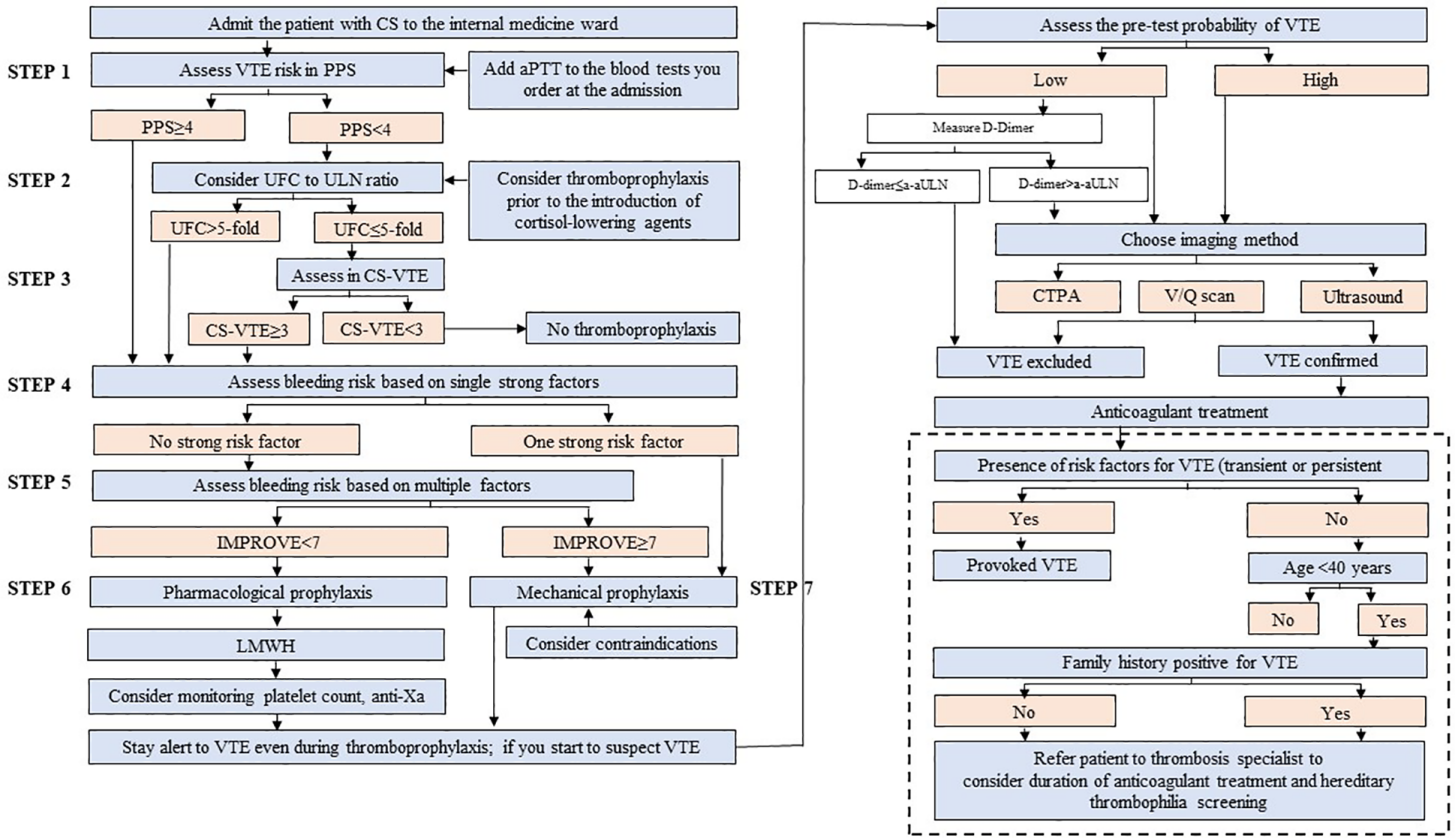
The algorithm for the thrombotic assessment in patients with Cushing’s syndrome (CS) not related to cancer, admitted to the internal medicine ward. VTE denotes venous thromboembolism; aPTT, activated partial thromboplastin time; PPS, Padua Prediction Score; UFC, urine free cortisol; ULN, upper limit of normal; CS-VTE, Cushing Syndrome Venous Thromboembolism score; IMPROVE, International Medical Prevention Registry on Venous Thromboembolism; LMWH, low-molecular-weight heparin; a-aULN, age-adjusted upper limit of normal; CTPA, computed tomography pulmonary angiogram; V/Q scan, ventilation/perfusion lung scintigraphy.

**Figure 3:**
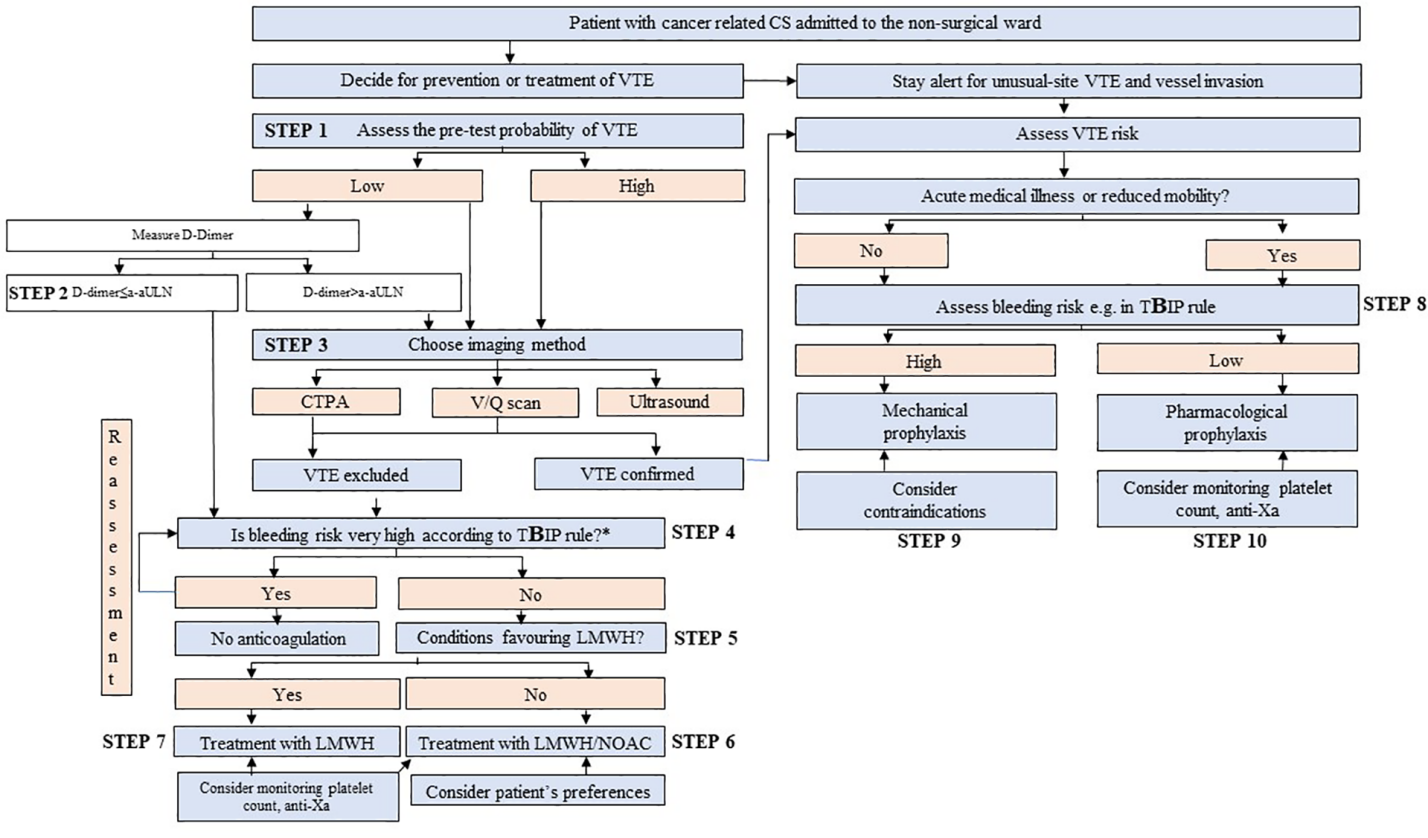
The algorithm for the thrombotic assessment in patients with cancer-related Cushing’s syndrome (CS). EAS denotes ectopic adrenocorticotropic hormone syndrome; VTE, venous thromboembolism; a-aULN, age-adjusted upper limit of normal; CTPA, computed tomography pulmonary angiogram; V/Q scan; ventilation/perfusion lung scintigraphy; TBIP, Thromboembolic risk, Bleeding risk, drug–drug Interactions, Patient preferences rule; LMWH, low-molecular-weight heparin; NOAC, non-vitamin K antagonist oral anticoagulant; ACC, adrenocorticotropic carcinoma. *Very high bleeding risk according to the European Society of Cardiology Guidelines on cardio-oncology: active or recent major bleeding (<1 month); recent/evolving intracranial lesions; platelet count <25 000/μL. According to the International Society on Thrombosis and Haemostasis, major bleeding is defined as: fall in hemoglobin level≥2 g/dL, transfusion of ≥2 units of red blood cells, fatal bleeding, or bleeding in a critical area (intracranial, intraspinal, intraocular, pericardial, intra-articular, intramuscular with compartment syndrome, or retroperitoneal).

**Figure 4:**
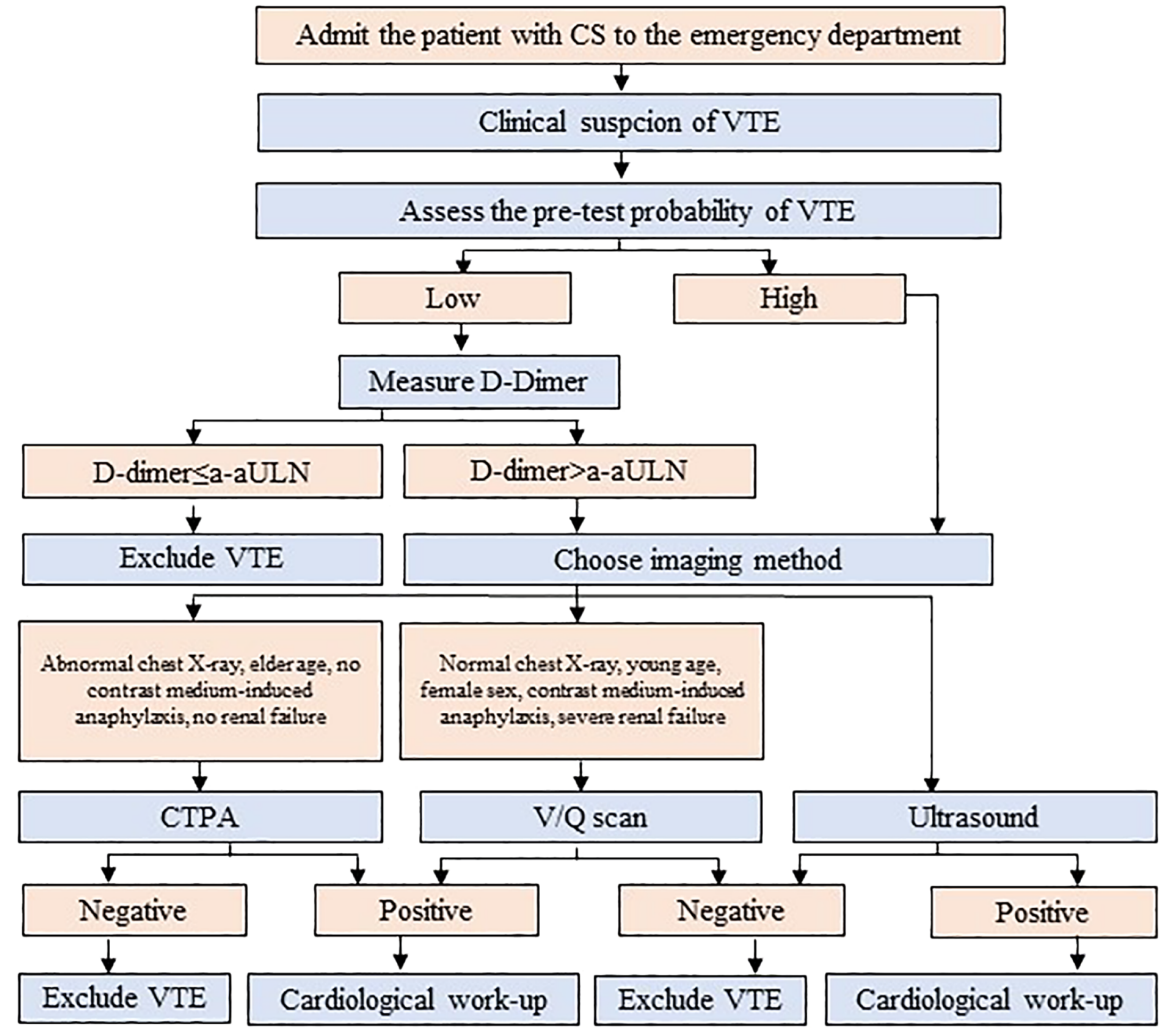
The algorithm for the diagnosis of venous thromboembolism (VTE) in patients with Cushing’s syndrome (CS) admitted to the emergency department. a-aULN denotes age-adjusted upper limit of normal; CTPA, computed tomography pulmonary angiogram; V/Q scan; ventilation/perfusion lung scintigraphy.

The algorithm to consider was presented in **Figure 1** and detailed in **Figures 2**–**4**.

### 3.1 Topic: risk factors for VTE

#### 3.1.1 Current practices

The three most frequently selected factors influencing the start of thromboprophylaxis in patients with CS are “previous VTE” (65%), “severity of hypercortisolism” (65%), and “limitation of mobility” (56%); other risk factors for VTE that influence the initiation of thromboprophylaxis include older age, cancer, and current smoking (43%); eight centers (35%) started thromboprophylaxis in all patients with CS regardless of the presence of risk factors (5).

#### 3.1.2 Question

An Endocrine Society clinical practice guideline suggests evaluating CS patients for risk factors of VTE (11). In light of current guidelines, which risk factors for VTE are to be assessed in the patients with CS hospitalized in the non-surgical wards?

#### 3.1.3 Response

##### 3.1.3.1 Classical risk factors for VTE

Previous VTE, limitation of mobility, older age, and cancer are well-established risk factors for VTE, incorporated into the Padua Prediction Score (further referred to as Padua Score) or International Medical Prevention Registry on Venous Thromboembolism (IMPROVE) score (27, 28) (**Table 1**). The patient with VTE and CS does not entirely embody the demographic risk factors that are acknowledged in Padua Score, although. One of the risk factors in Padua Score is age, awarded 1 point when it exceeds 70. The mean (or median age) when VTE occurs in patients with CS is far below 70 (10, 29), although this cutoff is used in publications referring only to patients with CS (29, 30). Moreover, the patient with CS and thrombosis is on average an overweight or obese patient (1 point for obesity in Padua Score); approximately one of six is on hormonal replacement therapy (estrogen or testosterone, 1 point in Padua Score) (10, 29). A patient with such characteristics would only be scored 1 or 2 points by the Padua Score and, therefore, not considered at high risk of VTE. Indeed, according to the Padua score, thromboprophylaxis (high risk of VTE in Padua Score ≥ 4 points) would be recommended in the presence of other risk factors, including previous VTE, immobilization, cancer, or an infection, the latter being recently shown to be a major cause of mortality in CS (31). Interestingly, Endo-ERN reference centers also based the decision to start thromboprophylaxis on the coexistence of these factors (5). During the process of developing the score devoted to patients with CS, CS-VTE score, a plethora of factors were checked for their association with VTE among patients with CS. The list included all factors from the Padua Score, with the exception of the previous VTE (29). The factors for VTE, such as obesity, estrogen treatment, trauma, recent surgery unrelated to CS, and active malignancy, were rejected from the final model as they did not gain the statistical significance (29), although they are commonly recognized risk factors for VTE.

**Table 1:** The comparison of the risk assessment scores for venous thromboembolism (27–29).

Score	PPS	Points	IMPROVE	Points	CS-VTE	Points
Factors	Reduced mobility (Bedrest with bathroom privileges (either due to patients’ limitations or on physicians order) for at least 3 days)	3	Immobilization ≥7 days	1	Reduced mobility (Bed rest with bathroom privileges for at least3 days)	2
	Active cancer (Patients with local or distant metastases and/or in whom chemo-therapy or radiotherapy had been performed in the previous 6 months)	3	Active cancer	2		
	Previous VTE, excluding superficial thrombophlebitis	3	Previous VTE	3		
	Known thrombophilic condition (Carriage of defects of anti-thrombin, protein C or S, factor V Leiden, G20210A prothrombin mutation, antiphospholipid syndrome)	3	Known thrombophilia (Defined as inherited or acquired disorder of hemostasis including antithrombin III deficiency, protein C deficiency, and protein S deficiency)	2		
	Recent trauma and/or surgery (<1 mo)	2				
	Elderly age (i.e., >70 y)	1	Age >60 y	1	Age ≥69 y	2
	Heart and/or respiratory failure	1				
	Acute myocardial infarction or ischemic stroke	1			Previous cardiovascular event (Acute myocardial infarction, ischemic stroke, transient ischemic attack)	1
	Ongoing hormonal treatment	1				
	Obesity (body mass index >30 kg/m^2^)	1				
	Acute infection and/or rheumatologic disorder	1			Acute severe infections	1
			Lower limb paralysis	2		
			ICU/CCU stay	1		
					Midnight plasma cortisol >3.15 ULN	1
					Shortened aPTT	1
Commentary	score ≥4 indicates high VTE risk		score ≥2 indicates high VTE risk		score ≥3 indicates high VTE risk	

The risk factors that are comparable to each other are shown in the same line (author’s suggestion).

PPS denotes Padua Prediction Score; Ps, points; IMPROVE, International Medical Prevention Registry on Venous Thromboembolism; IMPROVE, International Medical Prevention Registry on Venous Thromboembolism; CS-VTE, Cushing syndrome-venous thromboembolism; VTE, venous thromboembolism; ICU, intensive care unit; CCU, critical care unit; ULN, upper limit of normal; aPTT, activated partial thromboplastin time.

##### 3.1.3.2 CS-specific risk factors for VTE

The candidate factor that could be associated with the occurrence of VTE in young patients is the severity of hypercortisolemia. Severe hypercortisolism may be a life-threatening condition that mandates immediate treatment (11). Most of these patients have ectopic adrenocorticotropic hormone (ACTH) syndrome (EAS) associated with PE (11). Thromboprophylaxis is suggested in patients with urine free cortisol (UFC) >fivefold normal (especially if bedridden or with low mobility) (11). The data on the association of UFC with VTE are contradictory, confirming (32, 33) or denying (10, 29) this relationship. This holds true also for the association of UFC with the parameters of hemostasis, either positive (7, 34–36) or not correlated (2, 8, 35–39). Instead of UFC, the midnight plasma cortisol was incorporated into the CS-VTE score (29). The overall performance of the score was good, as it allowed to correctly discriminate the occurrence of VTE in 94% of the patients in the derivation cohort (29). The external validation is missing (29).

Some studies associated the introduction of cortisol-lowering medications with the occurrence of VTE (37, 40, 41). The authors speculated that the rapid fall in cortisol production may lead to the transient proinflammatory and procoagulant state, resulting in VTE (40). The data are ambiguous since, retrospectively, it has been speculated whether some of these complications were present prior to introducing the cortisol-lowering medications (37). Moreover, the pretreatment with cortisol-lowering agents reduced the risk of VTE after surgery (40) or exerted no effect (42). Finally, other studies showed no VTEs in patients treated with medication only (10).

The assessment of thrombotic risk does not mandate to stay alert to the clinical signs and symptoms of VTE (both PE and DVT), which should be diagnosed accordingly (right upper panel of **Figure 2**, details of the diagnostics presented in **Section 3.3.1**).

#### 3.1.4 Summary

To summarize, the algorithm to consider should contain the Padua Score, UFC, and CS-VTE (**Figure 2**). Such an approach (1) is in line with the most common approach of the specialty centers that deal with patients with CS and (2) provides a specific strategy that addresses both generic and CS-specific risk factors.

### 3.2 Topic: thrombophilia

#### 3.2.1 Current practices

Known hereditary thrombophilia and vWF promoter polymorphism haplotype 1 are used as criteria to decide the starting of thromboprophylaxis in patients with CS by 30% and 13% of centers, respectively. One of 25 reference centers (4%) reported to perform the hereditary thrombophilia screening routinely (5).

#### 3.2.2 Question

Which patients are screened for thrombophilia, and how this information may help in assessing VTE risk in patients with CS?

#### 3.2.3 Response

##### 3.2.3.1 Hereditary thrombophilia

The known hereditary thrombophilia was reported to be used in the decision to start thromboprophylaxis by 30% of reference centers of the Endo-ERN (5). In spite of the fact that the thrombophilia screening should not be performed to predict a first episode of VTE (23), the results of thrombophilia testing alter the VTE risk calculated in Padua Score (28), including acquired thrombophilia (23). The family history positive for VTE is categorized as an equal factor to the confirmed thrombophilia in the Caprini score for the general surgical patients (43). This should be treated with caution since none of the patients with CS and VTE, who were found to have thrombophilic defects [such as factor V Leiden (FVL), or prothrombin gene 20210A variant, or VWF gene promoter], had a positive family history of VTE (33).

In view of the current guidelines, testing for hereditary thrombophilia is to be considered (1) in young patients with either spontaneous VTE or VTE associated with weak environmental risk factors (**Table 2**) and (2) with a family history positive for VTE (3) if it can impact the management after the VTE episode, when balancing the risk of recurrence and contemplating cessation of the anticoagulation treatment (22, 23). Trying to identify the percentage of CS patients who might have had unprovoked VTE and, therefore, may need thrombophilia testing, we reviewed the available detailed description of the clinical situation of patients with CS and VTE (29, 40). No identifiable risk factors for VTE were reported for 15% (three of 20) of patients with CS and VTE analyzed by Zilio et al., but no patients were younger than 40 years (29). No identifiable risk factors for VTE were reported for 52.9% of patients with CS and VTE (nine of 19) (40). Among them, 33% (three of nine) were under 40 years, 44% (four of nine) were between 40–50 years (40). The information on family history of VTE is missing in both studies; therefore, the percentage of patients who might be candidates to thrombophilia screening is not clear, especially that it is recommended when it affects the management of VTE, meaning the cessation of anticoagulant treatment. The data on the recurrence of VTE after cessation of anticoagulation in patients with CS are scarce. Zilio et al. documented that 30% of patients had a second episode of VTE, after the one considered in their study (29). Such a percentage of patients with recurrent VTE correspond with unprovoked VTE (44). Currently, there are guidelines for the duration of anticoagulation for the prevention of recurrent VTE, devoted only to patients with CS. The duration of anticoagulation is decided at the discretion of the managing thrombosis specialist, and we can only suspect that it is individualized based on the sum of risk factors for VTE and hormonal status (CS remission/relapse).

**Table 2:** Examples of the factors that, when present in the given period of time prior to the venous thromboembolism (VTE) episode, mandate to categorize this VTE episode as provoked (25).

Examples of risk factors	Category
Surgery with general anesthesia for >30 minutes up to 3 months prior to VTE	Transient major
Surgery with general anesthesia for >30 minutes up to 2 months prior to VTE	Transient minor
Confined to bed in hospital (only ‘bathroom privileges’) for at least 3 days with an acute illness up to 3 months before VTE	Transient major
Confined to bed out of hospital for at least 3 days with an acute illness up to 2 months prior to VTE	Transient minor
Admission to hospital for less than 3 days with an acute illness up to 2 months prior to VTE	Transient minor
Leg injury associated with reduced mobility for at least 3 days up to 2 months prior to VTE	Transient minor
Cesarean section up to 3 months prior to VTE	Transient major
Pregnancy or puerperium up to 2 months prior to VTE	Transient minor
Estrogen therapy up to 2 months prior to VTE	Transient minor
Long haul flight up to 2 months prior to VTE	Transient minor
Active cancer	Persistent malignant
Inflammatory bowel disease	Persistent non-malignant
Active autoimmune disease	Persistent non-malignant

The prevalence of heterozygous FVL and prothrombin variant G20210A in patients with CS, was similar to that in the general population, regardless of the occurrence of VTE (10%–25% in case of VTE and 4%–6% without VTE) (29, 33), with no severe thrombophilias (i.e. antithrombin, protein C or protein S deficiency) (23). The activity assays are the mainstay of the diagnostic workup of both hereditary antithrombin and protein C deficiencies (26, 45). It is not clear, whether these assays give adequate results in patients with CS, who at baseline have been reported for elevated protein C, S, and antithrombin, when compared with control subjects (4).

##### 3.2.3.2 Von Willebrand factor promoter polymorphism haplotype 1

There are numerous reports on the elevation of vWF in patients with CS as compared with control subjects (4, 7, 46–50), comparison of vWF in active phase and after treatment (38, 48, 51), and the association between vWF promoter polymorphism with vWF level (35, 36, 52). The recent meta-analysis demonstrated no linear relationship between vWF level and number of thrombotic events (2); however, the logistic regression was not corrected for the blood type. When corrected for blood type, both vWF activity and antigen were elevated in patients with VTE, when compared with patients without VTE (29). However, neither vWF activity nor antigen was incorporated into CS-VTE risk assessment model (29). Instead, CS-VTE contained shorter activated partial thromboplastin time (aPTT) (29). The shortened aPTT has been postulated to result from the increased activity of many coagulation factors: II, V, VIII, IX, X, XI, XII, and vWF, (4, 7, 37, 47, 49, 50, 53–55); however, in most cases, elevated FVIII contributes to shortened aPTT in everyday practice. Since drawing blood for laboratory investigation often precedes the assessment in Padua Score during admission to the ward, it seems reasonable to perform a routine assessment of aPTT in each patient with CS, to have its result ready for the assessment in CS-VTE.

#### 3.2.4 Summary

In summary, (1) the routine screening for thrombophilia in patients with CS does not follow the current guidelines; (2) if the thrombophilia screening results are available, they affect the result of Padua Score; in this case, both hereditary and acquired thrombophilia should be considered. Among laboratory parameters that may help to better categorize patients with CS in terms of thrombotic risk, aPTT is more accessible than von Willebrand factor promoter polymorphism haplotype 1 in clinical practice. It seems to be more informative, since it is affected by numerous coagulation factors that have been reported to be affected in CS.

### 3.3 Topic: subtypes oF CS

#### 3.3.1 Current practices

Four centers (out of 23, 17%) investigated in the Endo-ERN consider the subtype of CS in the decision to start thromboprophylaxis. The prothrombotic-considered subtypes of CS most frequently named by these centers are EAS or ectopic corticotropin-releasing hormone syndrome (three of four), malignant adrenal CS (three of four), and, to a lesser extent, Cushing’s disease (CD) (one of four) (5).

#### 3.3.2 Question

How does the subtype of CS affect the assessment and management of thrombotic risk in CS?

#### 3.3.3 Response

Both patients with EAS and malignant adrenocortical cancer (ACC) require dedicated approach to their thromboembolic risk, presented below.

##### 3.3.3.1 Ectopic ACTH syndrome

The acute manifestation of thrombotic complications in EAS led to the concept of an emergency attitude for intense hypercortisolism (12). Due to the high prevalence of VTE among patients with EAS (up to 14%), thromboprophylaxis with heparin must be systematically prescribed (12), if the bleeding risk is acceptable (refer to R4.2 and **Supplementary No. 1**). Aside from severe hypercortisolism, the risk of VTE involves risk inherent with cancer (14, 19). The number of patients with EAS will already have PE at presentation and thus require anticoagulant treatment (12). Although PE prevailed over PE among patients with EAS (56), generally DVT is more prevalent than PE (with or without DVT) among patients with different types of cancer (57). Therefore, the diagnostics should encompass both DVT and PE. It may be hypothesized that a routine assessment toward VTE in all patients with EAS, based on the current guidelines developed by ESC (20), would ease the execution of the recommendations presented in this opinion (12) (**Figure 3**). The cornerstone of the diagnostics toward VTE includes assessment of the clinical pre-test probability (**Table 3**) (58, 59, 61), D-dimer (62) measurement, and computed tomographic pulmonary angiography (CTPA) result (20). The pre-test probability assessment is considered in the ESC guidelines to be a key step in all diagnostic algorithms for PE, and it has been internalized in the algorithm for CS patients (**Figure 3**; **Supplementary No. 2**). Analogical score is used for DVT (60). Assessing pre-test probability routinely in all patients with EAS might be considered a novel approach, as nowhere in the ESC guidelines is written that it should be routinely assessed in any group of patients. The plasma D-dimer is used to exclude PE in PE-unlikely patients, to reduce the need for unnecessary imaging and irradiation (20). There has been an attempt to identify the cutoff of D-dimer (≥2.6 μg/mL), which identified deep vein thrombosis (DVT) in a small study of 19 patients with overt or subclinical CS (63). In fact, the positive predictive value of elevated D-dimer levels is low and D-dimer testing is not useful for confirmation of PE or DVT (20). Moreover, its utility decreases in the hospitalized patients, patients with cancer, or with severe infection and inflammation, in whom more than 10 patients would have to be tested for D-dimer to exclude one PE. On the contrary, the respective number of patients in the general population of an emergency department is only three (20). Therefore, the separate algorithm refers to the patients with CS admitted to the emergency unit (**Figure 4**). Recently, it is recommended to consider adjusted D-dimer cutoff instead of the fixed cutoff level (age × 10 µg/L, for patients aged >50 years) (20). Since the positive correlation between D-dimer and age has been reported also in patients with CS (39), the age-adjusted cutoff should be considered in these patients.

**Table 3:** The clinical prediction rules for suspected venous thromboembolism (VTE) (20, 58–60).

VTE type	PE				DVT	
Score	Wells score	Simplified	Revised Geneva score	Simplified	Wells score	Original
Factors	Previous PE or DVT	1	Previous PE or DVT	1	Previously documented DVT	1
	Heart rate >100 b.p.m	1	Heart rate 75-94 b.p.m.	1		
			Hear rate ≥95 b.p.m.	2		
	Surgery or immobilization within the past 4 weeks	1	Surgery or fracture within the past month	1	Recently bedridden for ≥3 days, or major surgery within the previous 12 wks	1
	Haemoptysis	1	Haemoptysis	1		
	Active cancer	1	Active cancer	1	Active cancer	1
	Clinical signs of DVT	1	Unilateral lower-limb pain	1	Paralysis, paresis, or recent immobilization of the lower extremities	1
					Localized tenderness along the deep venous system	1
			Pain on lower-limb deep venous palpation and unilateral edema	1	Entire leg swollen	1
					Calf swelling ≥3 cm compared with the other side (measured 10 cm below tibial tuberosity)	1
					Pitting edema confined to the symptomatic leg	1
					Collateral superficial veins (nonvaricose)	1
	Alternative diagnosis less likely than PE	1			Alternative diagnosis at least as likely as DVT	1
			Age>65 years	1		
Clinical probability		0-1: unlikely; likely: ≥2		0-1: low; 2-4: intermediate; ≥5: high		0: low; 1-2: moderate; ≥3: high

The risk factors that are comparable to each other are showed in the same line (author’s suggestion).

PE denotes pulmonary embolism; DVT, deep vein thrombosis. The simplified scores were presented, when available.

The method of choice for imaging PE is multidetector CTPA (20), while the planar pulmonary ventilation/perfusion scintigraphy may preferentially be applied in selected patients (20). Although PE was the most common manifestation of VTE (9.3%, 4.6% fatal) in the patients with EAS (56), other thrombotic manifestations involved unprovoked DVT of axillary/subclavian veins and retinal vein thrombosis. The latter two belong to the unusual-site VTE, which clinicians dealing with EAS should stay alerted to. The state-of-the-art of the treatment of unusual-site VTE has been reviewed previously (64).

##### 3.3.3.2 Adrenocortical carcinoma

Approximately half of patients with ACC have clinical hormone excess (65). In these patients, just as in patients with EAS, the risk of VTE involves risk inherent with both hypercortisolism and cancer. There is no thromboembolic score devoted to hospitalized patients with cancer. The risk factors to consider are presented in **Table 4**. The predominant manifestation of VTE among patients with ACC is perioperative PE (23.5%, mostly during 10 weeks after surgery) (66). Nonetheless, the rate of PE not related to surgery reported in the retrospective study of patients with ACC was also high, that is, 5.9% (66). These two PEs not related to surgery encompassed episodes of PE occurred 1 day before surgery and 7 years prior to surgery (66). The extension of ACC into the adrenal vein, renal vein, or inferior vena cava occurs in approximately 15%–25% (65), and may reach the right atrium (67), or even cause saddle PE (68). The episode of VTE may precede the diagnosis of ACC, as presented in the case of a female patient in her 20s with an iliac vein DVT as an atypical presentation of subsequently diagnosed metastatic ACC (69).

**Table 4:** Risk factors for venous thromboembolism (VTE) in patients with cancer (21).

Patient-related factors	Ageing Comorbidities Sex (female) Hereditary coagulation defects Performance status Prior VTE history
Cancer-related factors	Cancer type Genetic characteristics (JAK2 or K-ras mutations) Histology (adenocarcinoma) Initial period after diagnosis Primary site (pancreas, stomach, ovaries,brain, lung, myeloma) Stage (advanced, metastatic)
Treatment-related factors	Cancer therapy Central venous catheters Hospitalization Major surgery

##### 3.3.3.3 Cushing’s disease

The increased prothrombotic profile in patients with CD when compared to adrenal CS has been attributed to the increased cortisol levels and vWF levels (34). The ACTH-dependent and -independent etiology of CS seems to similarly increase the VTE risk in the perioperative period, since the rates of VTE before treatment were similar in these two subtypes of CS (40).

#### 3.3.4 Summary

In conclusion, in the decision making whether to introduce thromboprophylaxis or not, the CS subtype should be taken into account, which is in line with the reviewed guidelines. The exclusion of PE at admission to the hospital ward may help to decide whether to introduce thromboprophylaxis or anticoagulation treatment in patients with EAS. Clinicians managing patients with cancer-related CS should stay alert for the symptoms of PE and DVT, unusual-site VTE, and invasion of tumors into the vessels (**Figure 3**).

### 3.4 Topic: LMWH

#### 3.4.1 Current practices

All 23 Endo-ERN reference centers that either routinely or selectively provided thromboprophylaxis to patients with CS reported LMWH as the first-choice anticoagulant drug for thromboprophylaxis in patients with CS (5). Non-vitamin K antagonists oral anticoagulants (NOAC) including apixaban, rivaroxaban, dabigatran, and edoxaban were not reported in this clinical setting (5).

#### 3.4.2 Question

Is LMWH efficient and safe in the prevention of VTE in patients with CS? Should the treatment be monitored? Is LMWH the only option for the treatment of VTE in cancer-related CS?

#### 3.4.3 Response

##### 3.4.3.1 LMWH in thromboprophylaxis in CS

There is compelling evidence that thromboprophylaxis with LMWH is highly effective and safe in medical patients hospitalized for acute medical diseases (13). However, data supporting such prophylactic strategy in patients with CS is sparse, and concerns mostly postoperative VTE (7, 8). The most alarming are the reports on the occurrence of VTE events in patients with CS while on thromboprophylaxis with LMWH (it also concerned perioperative setting) (10, 40, 41). It seems that LMWH should be equally effective in general population of patients with acute medical conditions and in patients with CS. Since the baseline risk of VTE in CS ranges from 1.5% to 14% (2, 3, 12), it is roughly comparable with the baseline VTE risk in medically ill patients (4.96%–14.9%) (70, 71). The mechanism of LMWH action indicates that it is useful in the clinical setting of CS. Although LMWH does not inactivate vWF (15), the key player in the hypercoaguability associated with CS, it targets the mutual point for the intrinsic and extrinsic pathways. Currently, there are no data showing that patients with CS should be administered with LMWH at alternative doses than the rest of the patients. In nonobese medical patients, LMWH is recommended to be administered in a fixed dose (15). According to the Recommendations on the Dosage of Anticoagulants in Obesity, enoxaparin 0.5 mg/kg once or twice daily may be considered in obese patients, but with the caution that evidence is only of a biochemical nature (72). No recommendations were made for dalteparin, although it has been concluded that a dose of 5,000 IE once daily may be insufficient in morbidly obese patients (72).

##### 3.4.3.2 Safety outcomes

The most common safety outcome of LMWH is bleeding. Pharmacological VTE prophylaxis in acutely or critically ill inpatients is recommended at acceptable bleeding risk, while mechanical prophylaxis, as described before, when bleeding risk is unacceptable (13). However, how to assess when the bleeding risk is unacceptable? Generally, the cumulative incidence of all kinds of bleedings (major and nonmajor in-hospital bleeding within 14 days of admission) was estimated at 3.2% (73). Such an incidence was observed among patients, older than the typical patient with CS (mean age 68.1 years), and with a median weight (69 kg) indicating much lower prevalence of obesity than among CS patients (73). Despite the differences in the clinical characteristics, it could be hypothesized that patients with CS can benefit from the IMPROVE bleeding risk assessment model (73) (**Table 5**). This model incorporates 11 predictors of bleeding, typically present in 10%–22% of assessed patients (score ≥7) (73–75). This score has not undergone extensive impact analyses showing their use leading to a reduction in clinical outcomes (13). An alternative approach is to analyze only three risk factors with the strongest association with bleeding: active gastroduodenal ulcer, bleeding in the 3 months before admission, and platelet count <50 × 103/l (17). In conclusion, the bleeding risk can be suspected when either one of the strongest risk factors is present, or when multiple risk factors coexist, as in the IMPROVE score (**Figure 4**). In case of high-bleeding risk, mechanical prophylaxis should be considered at high VTE risk patients (14), the method that could be limited by the fragile “tissue paper’ skin (76), that is, frequently observed in patients with CS (9) (**Supplementary No. 1**).

**Table 5:** The risk factors for in-hospital bleeding, included in the IMPROVE bleeding risk assessment score (74).

Score	Factor	Points
Factors	Renal failure (GFR 30-59 vs ≥60 ml/min/m^2^)	1
	Male vs female	1
	Age 40-80 vs < 40 y	1.5
	Current cancer	2
	Rheumatic disease	2
	Central venous catheter	2
	ICU/CCU stay	2.5
	Renal failure (GFR <30 vs >60 ml/min/m^2^)	2.5
	Hepatic failure (INR>1.5)	2.5
	Age ≥85 y vs <40 y	3.5
	Platelet count <50×10^9^/l	4
	Bleeding in 3 mo before admission	4
	Active gastroduodenal ulcer	4.5

Score ≥7 indicates high bleeding risk. Using these information may assist in deciding whether to use a pharmacological (score<7) or mechanical (score≥7) thromboprophylaxis. CCU denotes critical care unit; GFR, glomerular filtration rate; ICU, intensive care unit; INR, international normalized ratio; ULN, upper limit of normal; aPTT, activated partial thromboplastin time.

The most serious adverse non-bleeding reaction to LMWH is heparin-induced thrombocytopenia (HIT), which occurs mostly postoperatively if exposed to unfractionated heparin, and in cardiac surgery patients (18). In patients at risk of developing HIT, the platelet number should be monitored (18). In patients positive for HIT in the past, fondaparinux 2.5 mg. s.c. should be used instead of LMWH. The evidence for fondaparinux efficacy is scarce but shows 47% relative risk reduction in VTE versus placebo, with no increase in the major bleeding (77). Some experts suggest monitoring with anti-Xa activity at 4h after administration in obese patients and in those with renal insufficiency (creatinine clearance, CrCl ≤30 ml/min) (15). The literature showing the correlation of the LMWH dose with anti-Xa level has been summarized in (15). For patients with a CrCl ≤30 mL/min who require pharmacologic VTE prophylaxis, the manufacturer of enoxaparin recommends that 30 mg once daily be used (15).

##### 3.4.3.3 Other drugs in the thromboprophylaxis and other applications of LMWH for patients with CS

The high-risk outpatients [Khorana score of at least ≥2 (78)] with cancer may be offered thromboprophylaxis with not only LMWH but also apixaban [2.5 mg twice daily orally (79)], rivaroxaban [10 mg once daily orally (80)], provided that there are no significant risk factors for bleeding and no drug interactions (19). An attempt to extrapolate this recommendation to patients with ACC treated with mitotane is challenging. First, one of the predictor in Khorana score to assess the risk is site of the tumor (78), and it is not clear which category the patients with ACC are to be classified. Second, the product characteristics informs that one of the very common undesirable effect of mitotane is prolonged bleeding. The thromboprophylaxis with apixaban resulted in increased rate of major bleeding as compared with placebo (79), while with rivaroxaban with comparable bleeding risk as compared to placebo (80), while the head-to-head comparison with LMWH is not available.

The other application of LMWH, other than thromboprophylaxis, is cancer-related VTE, although recently physicians have reached for non-vitamin K antagonists anticoagulants (NOAC) to treat patients with cancer-related VTE (67, 69). No tool is currently available to predict the risk of bleeding episodes in this patient with cancer (81). In the algorithm to consider, the structured approach following the TBIP (Thromboembolic risk, Bleeding risk, Interactions, Patient preferences) rule was internalized (21). If negative for very high-bleeding risk, the patient should start anticoagulant treatment, and the choice between LMWH and NOAC is to be made (21). Conditions favoring LMWH involve NOAC major drug–drug interactions (21), the latter one available to check in the review (81) (**Supplementary No. 2**).

#### 3.4.4 Summary

In summary, (1) LMWH is the mainstay of thromboprophylaxis in patients with CS, with the need for platelet count or anti-Xa monitoring in selected patients; (2) bleeding risk can be assessed using multivariate approach or based on the presence of a single factor; (3) NOACs have been studied in thromboprophylaxis in high-risk outpatients with cancer, but the extrapolation to patients with ACC is challenging due to the paucity of data; and (4) NOAC may be used instead of LMWH in the treatment of cancer-related VTE after careful consideration.

## 4 Discussion

This article reviews the current state of art on the thromboprophylaxis in CS, in the light of the current guidelines for hospitalized patients. Our article is the first to present an algorithm to consider in the thrombotic risk assessment among patients with CS, as a starting point for a broader discussion in the environment. Combining current practices with the review of guidelines referring to hospitalized patients resulted in an interesting perspective on the topic, with an emphasis on the practical aspects of managing the thrombotic complications of CS.

We have identified uncertainties at every step of the algorithm preparation, that is, the appraisal of VTE risk and thrombophilia, in the different types of CS, and the therapeutic use of LMWH. Some points that need to be raised have already been discussed in the Results section, for example, the lack of validation of the CS-VTE score that has been incorporated into the algorithm. On the other hand, validated scores, such as the Padua Score or IMPROVE, are not entirely supported in the literature as applicable for all patient cohorts (82). The strength of the evidence that supports the algorithm is not graded, but is mostly low, or absent, and relies upon the expert’s opinion, as the introduction of the thromboprophylaxis in patients with UFC exceeding 5 times the ULN, as described in the Endocrine Society guidelines (11).

Another important consideration is related to the shortened aPTT, one of the criteria in CS-VTE score to decide whether to introduce thromboprophylaxis or not in patients with CS. Since the shortened aPTT may also be the result of preanalytical mistakes, in doubtful cases, it is advisable to contact the laboratory and repeat the aPTT test (83), before deciding to introduce thromboprophylaxis based on the result of a shortened aPTT. However, the association between a shortened aPTT and the risk of VTE has been evidenced in situations different from CS (84). Moreover, in CS-VTE score, aPTT is used as one of five criteria, not as a single factor in the decision-making process. Another important consideration while preparing our algorithm was the congruency between the clinical characteristics of the patients with CS and the cohorts included in the studies that targeted the assessment of VTE risk and management. If congruency were high, the methods of assessment, diagnosis, and treatment could be extrapolated to patients with CS. Taken together, we consider the algorithm as a starting point for a broader discussion in the context of CS.

We strongly believe that the uncertainties, present at every phase of the algorithm preparation, stem from insufficient knowledge of the course of CS-related VTE, with an emphasis on the rate of recurrence. This lack of evidence is directly related to the rarity of CS in the general population, which translates into low numbers of patients with CS and VTE, despite the increased risk of VTE in patients with CS. In fact, the rate of VTE recurrence allows categorizing factors predisposing to VTE into “major transient” and “minor transient.” (25) By definition, a risk factor is considered “major’ if it has been shown to be associated with (1) half the risk of recurrent VTE after stopping anticoagulant therapy (compared to no transient risk factor), when the risk factor occurred up to 3 months before the VTE, or (2) a greater than 10-fold increase in the risk of having a first VTE (25). In fact, VTE provoked by a transient factor has a low risk for a recurrent VTE, as opposed to a VTE provoked by a persistent risk factor, while the risk of recurrence in idiopathic VTE is somewhere in the middle (25). The duration of secondary prevention for recurrent VTE is decided on the discretion of the consulting hematologist/thrombosis specialist (85). The simplified approach to this decision has been presented in **Figure 2** and depends on the presence of the factors predisposing to VTE.

Although VTE risk is increased in CS to a comparable extent (>10 times) to that observed when other major factors predisposing to VTE are present, the risk of recurrent VTE in patients with CS is not established. Moreover, due to the high risk of persistent and recurrent CS (11, 86), the transient character of this factor may be questionable. Even in patients with Cushing’s disease with biochemical remission after transsphenoidal surgery, hypercoagulability is detected for at least 6 months post-surgery, as evidenced by an increased D-dimer level when compared to control patients (39). In light of current guidelines, the time of secondary prevention of recurrent VTE among patients with CS, without other predisposing factors, is not clear. Considering the presence of persistent or recurrent hypercortisolemia in the decision-making process, discontinuing anticoagulation after primary treatment is currently not recommended in any guidelines; however, it seems necessary.

To sum up, a plethora of factors affect VTE risk in patients with CS. Given the paucity of evidence, the proposed algorithm should be considered with caution and subjected to a broader discussion, when further evidence becomes available.

## Data availability statement

The original contributions presented in the study are included in the article/**Supplementary Material**, further inquiries can be directed to the corresponding author/s.

## Ethics statement

Ethical approval was not required for the study involving humans in accordance with the local legislation and institutional requirements. Written informed consent to participate in this study was not required from the participants or the participants’ legal guardians/next of kin in accordance with the national legislation and the institutional requirements.

## Author contributions

AB-W: Writing – original draft, Conceptualization, Data curation. MM: Writing – review & editing. RŚ-S: Writing – review & editing. AU: Writing – review & editing. AH-D: Writing – review & editing. SW: Writing – review & editing. EV: Writing – review & editing. AG-J: Conceptualization, Writing – review & editing.
