# Supplementary material for: The thrombotic risk in Cushing’s syndrome—questions, answers, and the algorithm to consider in its assessment: part I—thrombotic risk not related to surgery
Source: Front Endocrinol (Lausanne). 2024 Mar 11;15:1350010. doi: 10.3389/fendo.2024.1350010 (PMC10961355; doi:10.3389/fendo.2024.1350010)
Supplement: Supplementary file 1 [file DataSheet_1.docx]

**RISK ASSESSMENT FOR VENOUS THROMBOEMBOLISM (VTE) IN PATIENTS WITH CUSHING’S SYNDROME (CS)**

**Step one**

In step one, assess patient admitted for medical reasons using the Padua Prediction Score (PPS). Award the suitable number of points for the admitted patient. Proceed to the next step if the result is <4. Proceed to step four if the result is ≥4.

| PPS | Points | Patient |
| --- | --- | --- |
| Reduced mobility  Bedrest with bathroom privileges, either due to patients limitations or on physicians order, for at least 3 days) | 3 |  |
| Active cancer  (Patients with local or distant metastases and/or in whom chemo-therapy or radiotherapy had been performed in the previous 6 months) | 3 |  |
| Previous VTE, excluding superficial thrombophlebitis | 3 |  |
| Known thrombophilic condition  Carriage of defects of anti-thrombin, protein C or S, factor V Leiden, G20210A prothrombin mutation, antiphospholipid syndrome | 3 |  |
| Recent trauma and/or surgery (<1 mo) | 2 |  |
| Elderly age (i.e., >70 y) | 1 |  |
| Heart and/or respiratory failure | 1 |  |
| Acute myocardial infarction or ischemic stroke | 1 |  |
| Ongoing hormonal treatment  (Ongoing use of hormone-replacement therapy or oral contraceptives) | 1 |  |
| Obesity (body mass index >30 kg/m^2^) | 1 |  |
| Acute infection and/or rheumatologic disorder | 1 |  |

**Step two**

In step two, assess patients that have been awarded <4 points in PPS. Proceed to the step three if the result is ≤5. Proceed to the step four if the result is >5.

| Urine free cortisol (UFC) |  |
| --- | --- |
| Upper limit of normal (ULN) |  |

$\frac{UFC}{ULN}$=

**Step three**

In step three, assess only patients who had the UFC/ULN ratio ≤5. Proceed to the step four if the result is ≥3. Stop there if the result is <3.

| CS-VTE | Points | Patient |
| --- | --- | --- |
| Reduced mobility  (Bed rest with bathroom privileges for at least3 days) | 2 |  |
| Age ≥69 y | 2 |  |
| Previous cardiovascular event  (Acute myocardial infarction, ischemic stroke, transient ischemic attack) | 1 |  |
| Acute severe infections | 1 |  |
| Midnight plasma cortisol >3.15 ULN | 1 |  |
| Shortened activated partial thromboplastin time | 1 |  |

**Step four**

In step four, assess patients who had: PPS ≥4, or the UFC/ULN ratio >5, or CS-VTE ≥3. Proceed to the next step if none of these is true. Proceed to the step seven if any of these is true.

| Single strong risk factor | Tick |
| --- | --- |
| Active gastroduodenal ulcer |  |
| Bleeding in 3 months before admission |  |
| Platelet count <50×10^3^/l |  |

**Step five**

In step five, assess patients who did not have any of the single strong risk factors for bleeding. Proceed to the next step if the IMPROVE score is <7. Proceed to step seven if IMPROVE ≥7.

| IMPROVE score | Points | Patient |
| --- | --- | --- |
| Renal failure (glomerular filtration rate [GFR] 30-59 vs ≥60 ml/min/m^2^) | 1 |  |
| Male sex | 1 |  |
| Age 40-80 y | 1.5 |  |
| Current cancer | 2 |  |
| Rheumatic disease | 2 |  |
| Central venous catheter | 2 |  |
| Stay in the intensive care unit or critical care unit | 2.5 |  |
| Renal failure (GFR <30 vs >60 ml/min/m^2^) | 2.5 |  |
| Hepatic failure (International Normalized Ratio >1.5) | 2.5 |  |
| Age ≥85 y | 3.5 |  |
| Platelet count <50×10^9^/l | 4 |  |
| Bleeding in 3 mo before admission | 4 |  |
| Active gastroduodenal ulcer | 4.5 |  |

**Step six**

In step six, introduce pharmacological thromboprophylaxis with low-molecular-weight heparin in patients who are negative for past history for heparin-induced thrombocytopenia in the past (that may occur especially in patients who had cardiosugery). If the latter is false, use e.g. fondaparinux.

**Step seven**

In step seven, introduce mechanical thromboprophylaxis with either graduated compression stockings, or pneumatic compression devices. Do not offer anti-embolism stockings to people who have:

| Finding | Tick |
| --- | --- |
| Suspected or proven peripheral arterial disease |  |
| Peripheral arterial bypass grafting |  |
| Peripheral neuropathy or other causes of sensory impairment |  |
| Any local conditions in which anti-embolism stockings may cause damage – for example, fragile 'tissue paper' skin, dermatitis, gangrene or recent skin graft |  |
| Known allergy to material of manufacture |  |
| Severe leg oedema |  |
| Major limb deformity or unusual leg size or shape preventing correct fit |  |
