# Supplementary material for: The thrombotic risk in Cushing’s syndrome—questions, answers, and the algorithm to consider in its assessment: part I—thrombotic risk not related to surgery
Source: Front Endocrinol (Lausanne). 2024 Mar 11;15:1350010. doi: 10.3389/fendo.2024.1350010 (PMC10961355; doi:10.3389/fendo.2024.1350010)
Supplement: Supplementary file 2 [file DataSheet_2.docx]

**RISK ASSESSMENT FOR VENOUS THROMBOEMBOLISM (VTE) IN PATIENTS WITH ECTOPIC ADRENOCORTICOTROPIC (ACTH) HORMONE SYNDROME (EAS)**

**Step one**

To decide, whether to introduce the preventive or therapeutic dose of low-molecular-weight heparin (LMWH), exclude pulmonary embolism (PE). Assess the patient with EAS admitted to the non-surgical ward using one of the simplified pre-test probability of PE and DVT using e.g. simplified Wells score for PE and Wells score for DVT. You may use simplified Geneva risk score instead; or any of the latter two scores in the original form, available online. Award one point for each tick in the table. Proceed to the step two if the result is<2 for PE or 0 for DVT, and if you decide to measure D-dimer. However, keep in mind that D-dimer is frequently elevated in patients with cancer.

Proceed to the step three if you decide to bypass the D-dimer measurement, or if the simplified Wells rule for PE is ≥2; or Wells rule for DVT is ≥1 (and in this case, it is ≥1 due to the active cancer).

| The simplified Wells rule for PE | Tick |
| --- | --- |
| Previous PE or deep vein thrombosis |  |
| Heart rate >100 b.p.m |  |
| Surgery or immobilization within the past 4 weeks |  |
| Haemoptysis |  |
| Active cancer |  |
| Clinical signs of deep vein thrombosis |  |
| Alterative diagnosis less likely than PE |  |

| The Wells rule for DVT | Tick |
| --- | --- |
| Previously documented DVT |  |
| Recently bedridden for ≥3 days, or major surgery within the previous 12 wks |  |
| Active cancer |  |
| Paralysis, paresis, or recent immobilization of the lower extremities |  |
| Localized tenderness along the deep venous system |  |
| Entire leg swollen |  |
| Calf swelling ≥3 cm compared with the other side (measured 10 cm below tibial tuberosity) |  |
| Pitting edema confined to the symptomatic leg |  |
| Collateral superficial veins (nonvaricose) |  |
| Alternative diagnosis at least as likely as DVT |  |

**Step two**

In step two, measure D-dimer. Consider using age-adjusted D-dimer cut-off to exclude PE instead of fixed D-dimer cut-off (500 µg/l). If the D-dimer is within the used cut-off, proceed to the step eight. If the D-dimer is over the used cut-off, proceed to the step three.

The age-adjusted D-dimer cut-off: $Age\times10 \frac{\mu g}{l}$ for patients >50 year-old

**Step three**

Choose the imaging method. Use the ultrasound for suspected DVT. For PE diagnostics, use preferentially computed tomography pulmonary angiogram, especially in abnormal chest X-ray, elder age, no contrast medium-induced anaphylaxis, and no renal failure. Use the planar ventilation/perfusion lung scintigraphy in young, particularly female patients, in patients with contrast medium-induced anaphylaxis, or severe renal failure. Note that performing only a perfusion scan might be acceptable in patients with a normal chest X-ray; any perfusion defect in this situation would be considered a mismatch. Proceed to the step eight if the imaging is negative for DVT. Proceed to the step four if the imaging is positive for DVT.

**Step four**

Assess bleeding rule using TBIP (Thromboembolic risk, Bleeding risk, drug–drug Interactions, Patient preferences) rule. Consider the following risk factors: thrombocytopaenia, gastrointestinal or genitourinary cancer, gastrointestinal comorbidities, or gastrointestinal toxicity, recent or evolving intracranial lesions, active bleeding or recent major bleeding, severe renal dysfunction (estimated glomerular filtration rate [GFR] <30 mL/min/1.73 m2). Assess toward the very high bleeding risk that is associated with the factors presented below. If the risk of bleeding is very high, the management of patients with VTE should be individualized by the multidisciplinary team; the anticoagulation should not be introduced. Reassess the patient in terms of bleeding risk, to decide whether the bleeding risk is no longer very high, and the anticoagulation may be introduced. If there are no factors for the very high bleeding risk, proceed to step five.

|  | Tick |
| --- | --- |
| Active or recent major bleeding (<1 month)  According to the International Society on Thrombosis and Haemostasis, major bleeding is defined as: fall in hemoglobin level≥2 g/dL, transfusion of≥2 units of red blood cells, fatal bleeding, or bleeding in a critical area (intracranial, intraspinal, intraocular, pericardial, intra-articular, intramuscular with compartment syndrome, or retroperitoneal) |  |
| Recent/evolving intracranial lesions |  |
| Platelet count <25 000/μL |  |

**Step five**

Consider factors favoring LMWH in the treatment of VTE. If any of these is true, proceed to step seven. If none of these is true, proceed to step six.

|  | Tick |
| --- | --- |
| Unoperated gastrointestinal/genitourinary cancer |  |
| Gastrointestinal comorbidities or gastrointestinal toxicity |  |
| Severe renal dysfunction (creatinine clearance <15 mL/min); |  |
| Non-vitamin K antagonist oral anticoagulants (NOACs) major drug–drug interactions |  |
| Platelet count <50 000/μL |  |

**Step six**

Apixaban, edoxaban, or rivaroxaban are recommended for the treatment of symptomatic or incidental VTE in patients with cancer without contraindications, presented in the table. Note that the patients with primary brain tumours or brain metastases and acute leukaemia were excluded from the seminal apixaban trial.

The chosen, significant drug-drug interactions, essential to physician managing patients with CS, are listed below.

| Contraindications to the treatment with NOACs | Tick |
| --- | --- |
| High risk of gastrointestinal or genitourinary bleeding |  |
| Gastrointestinal absorption concerns |  |
| Significant drug–drug interactions |  |
| Severe renal dysfunction (CrCl,15 mL/min) |  |
| Significant liver disease (alanine aminotransferase/aspartate aminotransferase > 2×upper limit of normal |  |
| Significant thrombocytopaenia (platelet count<50 000/μL) |  |

| Chosen cancer-related therapy | Cytochrome p450CYP3A4 | P-glycoprotein |
| --- | --- | --- |
| Mitotan | ↑ |  |
| Methylprednisolone | ↑ |  |
| Dexamethasone | ↑ | ↑ |
| Etoposide | ↓ | ↑ |
| Doxorubicin | ↓ | ↑ |

**Step seven**

Introduce LMWH in the therapeutic dose. In patients with cancer with platelet counts of 25 000–50 000/μL, anticoagulation with half-dose LMWH may be considered after a multidisciplinary discussion.

**Step eight**

Patients with EAS are at increased risk of VTE. If the risk of bleeding is tolerable, and PE excluded, introduce LMWH in the prophylactic dose. No tool is currently available to predict the risk of bleeding episodes in this patient with cancer. In the decision whether to introduce thromboprophylaxis, consider the factors outlined below. If the risk of bleeding is tolerable, go to step nine. Otherwise, go to step ten.

**Step nine**

Introduce the pharmacological thromboprophylaxis with low-molecular-weight heparin in patients who are negative for the past history for heparin-induced thrombocytopenia in the past (that may occur especially in patients who had cardiosugery). If the latter is false, use e.g. fondaparinux.

**Step ten**

In step seven, introduce the mechanical thromboprophylaxis with either graduated compression stockings, or pneumatic compression devices. Do not offer anti-embolism stockings to people who have:

| Finding | Tick |
| --- | --- |
| Suspected or proven peripheral arterial disease |  |
| Peripheral arterial bypass grafting |  |
| Peripheral neuropathy or other causes of sensory impairment |  |
| Any local conditions in which anti-embolism stockings may cause damage – for example, fragile 'tissue paper' skin, dermatitis, gangrene or recent skin graft |  |
| Known allergy to material of manufacture |  |
| Severe leg oedema |  |
| Major limb deformity or unusual leg size or shape preventing correct fit |  |
